# Advancing Optical Techniques for Cancer Diagnosis and Therapy Monitoring

**DOI:** 10.1117/1.BIOS.2.4.042701

**Published:** 2025-12-13

**Authors:** Javier A. Jo, Narasimhan Rajaram, Srivalleesha Mallidi, Alexandra Walsh

**Affiliations:** a The University of Oklahoma, Gallogly College of Engineering, School of Electrical and Computer Engineering, Norman, Oklahoma, United States; b University of Arkansas, College of Engineering, Department of Biomedical Engineering, Fayetteville, Arkansas, United States; c Tufts University, School of Engineering, Department of Biomedical Engineering, Medford, Massachusetts, United States; d Texas A&M University, College of Engineering, Department of Biomedical Engineering, College Station, Texas, United States

## Abstract

The editorial comments on the BIOS Special Section on Optics for Cancer Therapy Monitoring and Diagnosis, which published as a series in Volume 2.

The biophotonics community has continued to explore the potential of novel optical technologies for cancer diagnosis and monitoring response to therapy. Today, these technologies are not only advancing fundamental science but also addressing critical clinical challenges. Innovations that enable deeper imaging, novel contrast mechanisms, and real-time feedback are transforming how we evaluate cutting-edge treatments—from targeted agents and immunotherapies to radiation and phototherapies. In many ways, biophotonic technologies are a natural fit because these techniques are highly sensitive to changes in the tumor microenvironment that drive progression, invasion, and resistance to therapy. Such microenvironmental changes include changes in cell density, angiogenesis, tissue and vascular hypoxia, cell metabolism, extracellular matrix remodeling, and immune cell infiltration. This sensitivity has allowed biophotonic approaches to also reveal new dimensions of cancer biology, offering insights that may shape the next generation of therapies.

The BIOS special section “Optics for Cancer Therapy Monitoring and Diagnosis,” edited by Javier A. Jo, Narasimhan Rajaram, Srivalleesha Mallidi, and Alexandra Walsh, brings together research at the forefront of this progress. This collection includes articles focused on cancer diagnosis as well as the identification of long-term outcomes. Grajales et al. (10.1117/1.BIOS.2.3.032706) discuss the development of a convolutional neural network (CNN) developed on Raman spectroscopy-based features from 202 ex vivo prostate, breast, and brain tumors. This CNN was then applied prospectively on a small cohort of 10 patients to determine the ability to distinguish normal tissue from prostate cancer in real-time and achieved an area under the receiver operative characteristic curve of 0.76, supporting the continued translation of Raman-spectroscopy-based tools for intraoperative decision-making.

Lagarto and colleagues (10.1117/1.BIOS.2.3.032705) present results from a clinical study to determine if autofluorescence imaging based on excitation at 375 and 445 nm can accurately differentiate between benign and malignant tissue. Using a supervised ensemble learning model built on data from 73 patients and tested on 44 patients, they were able to achieve a sensitivity of 85.2% and specificity of 84.5%.

Our third and final cancer diagnosis paper by Bonaventura et al. (10.1117/1.BIOS.2.2.022704) is also a clinical study that explores whether autofluorescence, hyperspectral, optical coherence tomography, and polarized light imaging systems can provide complementary sources of information that help differentiate between healthy and cancerous esophageal tissue samples acquired during upper endoscopy. This study was performed in 45 samples acquired from 26 patients and showed that polarized light imaging provided the strongest discriminatory power.

This collection also includes two articles that explore the ability of optical techniques to provide information about tumor outcomes, such as metastasis and treatment resistance. Elias et al. used second harmonic generation (SHG) microscopy to investigate the extracellular matrix in formalin-fixed paraffin embedded samples obtained from patients with colorectal adenocarcinoma and invasive ductal carcinoma (10.1117/1.BIOS.2.2.022703). Specifically, their study investigated whether fiber angle variability and forward-to-backward SHG scattering ratio (F/B), which has already been shown to be prognostic of metastasis-free survival in previous studies, varied with race. Their results show that F/B ratio obtained from the tumor-stroma interface was significantly different between demographic groups in invasive ductal carcinoma, demonstrating the need for algorithms based on such biomarkers to accurately account for race.

Finally, Yan et al. used exogenous probes to label and determine metabolic differences between radiation-resistant and radiation-sensitive cancer cells (10.1117/1.BIOS.2.1.012702). Using two exogenous probes—2-NBDG, to report glucose uptake, and TMRE, to determine mitochondrial membrane potential—they found increased glucose uptake in radioresistant head and neck cancer cells that could be linked to activation of hypoxia-inducible factor (HIF-1), a master regulator of oxygen homeostasis and a promoter of radiation resistance. These studies show the potential of using low-cost optical technologies to identify possible treatment resistance in cancer cells.

Overall, this collection of articles reflects the current state of the field and point toward exciting future directions. We invite you to explore these contributions:

• “Deployment of a real-time prostate cancer confirmation system with Raman spectroscopy: fine-tuning versus test-time adaptation of 1D CNNs” by David Grajales, William Le, Victor Blanquez-Yeste, Frédérick Dallaire, Feryel Azzi, Guila Delouya, Dominique Trudel, Frédéric Leblond, Cynthia Ménard, and Samuel Kadoury
• “Identification of colorectal malignancies enabled by phasor-based autofluorescence lifetime macroimaging and ensemble learning” by João Lagarto, Alberto Ignacio Herrando, Rafaela Rego, Laura Fernández, José Azevedo, Hugo Domingos, Pedro Vieira, Amjad Parvaiz, Vladislav Shcheslavskiy, Pedro Silva, and Mireia Castillo Martin
• “Multimodal esophageal cancer imaging: establishing data processing techniques and assessing diagnostic sensitivity” by Justina Bonaventura, Natzem Lima, Joshua Routh, Aws Alameri, Shivanand Bomman, Bhaskar Banerjee, Hemanth Gavini, and Travis Sawyer
• “Exploring racial differences in second-harmonic-generation–based prognostic indicators of metastasis in breast and colon cancer” by Tresa Elias, Danielle Desa, Edward Brown IV, Showmick Paul, Gabriel Ramirez, Bradley Turner, Kelley Madden, Raul Gonzalez, Anna Weiss, and Edward Brown III
• “Optical imaging provides flow-cytometry–like single-cell level analysis of HIF-1α-mediated metabolic changes in radioresistant head and neck squamous carcinoma cells” by Jing Yan, Carlos Frederico Goncalves, Pranto Soumik Saha, Cristina Furdui, and Caigang Zhu

